# Myocardial infarct delineation *in vivo* using diffusion tensor MRI and the tractographic propagation angle

**DOI:** 10.1186/1532-429X-15-S1-P2

**Published:** 2013-01-30

**Authors:** Choukri Mekkaoui, Shuning Huang, Guangping Dai, Timothy G Reese, Jeremy Ruskin, Udo Hoffmann, Marcel P Jackowski, David E Sosnovik

**Affiliations:** 1 Radiology, Harvard Medical School - Massachusetts General Hospital, Boston, MA, USA

## Background

Delayed gadolinium enhancement (Gd-DE) is widely used to detect scar formation following myocardial infarction (MI) [1], but cannot be performed in patients with renal impairment. Here we use the tractographic propagation angle (PA), a novel index derived from 3D diffusion tensor MRI (DTI), to detect changes in myocardial fiber architecture post-MI [2]. We compare image segmentation based on the tractographic PA to infarct delineation with Gd-DE.

## Methods

**Figure 1:**
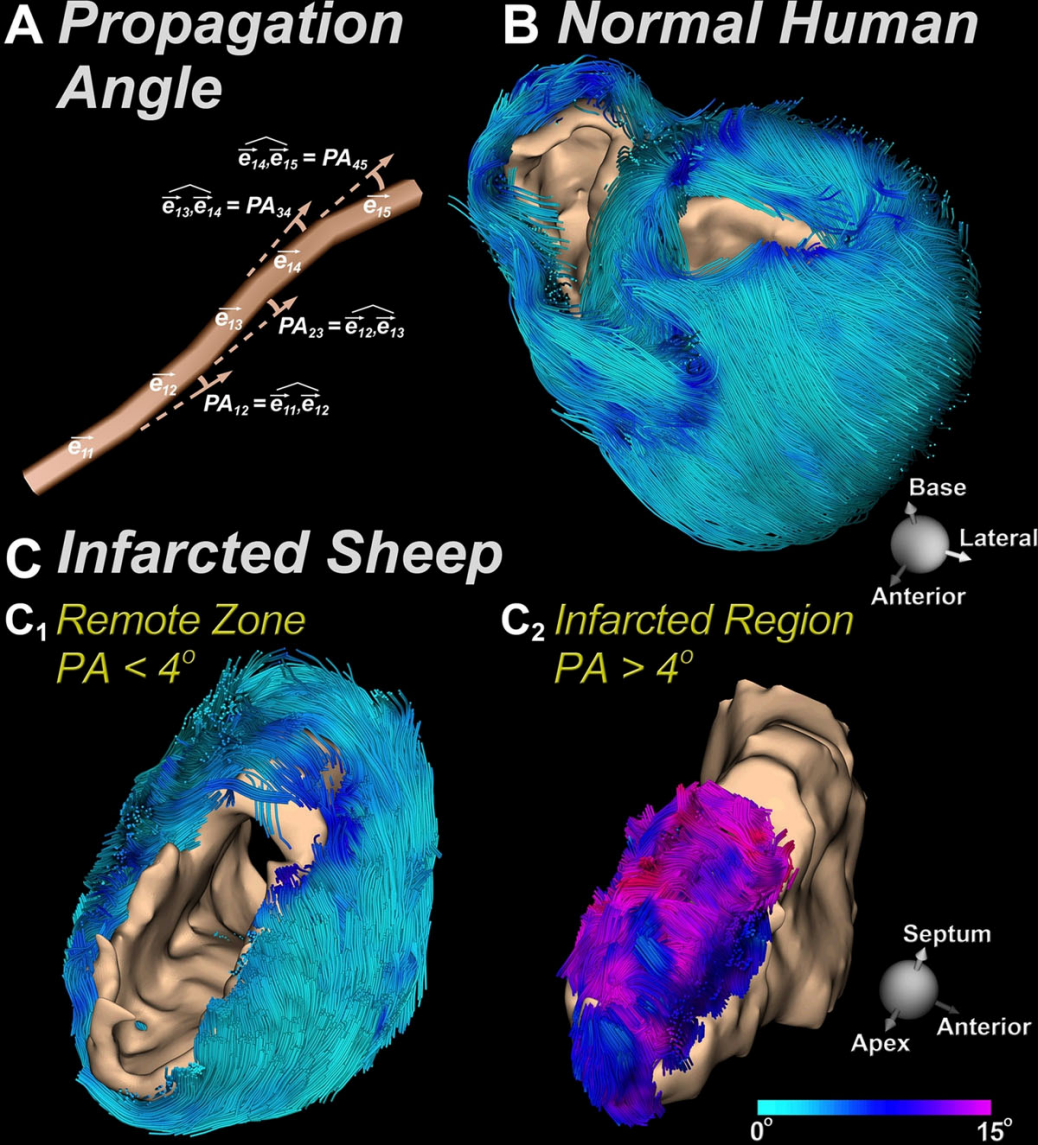
Tractograms color-coded by the propagation angle (PA). (A) PA is defined as the angle between two adjacent principal eigenvectors (ê_ij_, ê_ij+1_) relative to a given myofiber. (B) Normal human heart viewed from the base, showing a low and homogenous PA. (C) Sheep heart with a large anteroseptal infarct. (C1) A low-pass PA value of 4 degrees delineates the normal myocardium and creates a void in the infarct. (C2) Conversely, a high-pass PA value of 4 degrees robustly delineates the infarcted myocardium.

Normal human (n=5) and infarcted sheep hearts (n=6) were studied *ex vivo*. Infarcted mice (n=7) were imaged *in vivo*. MI was produced in C57BL6 mice via permanent ligation of the left coronary artery. *In vivo* DTI was performed on a 9.4T scanner (Bruker) using a 3D fat-suppressed single-shot 3D spin echo EPI sequence with motion-compensated diffusion-encoding gradients in 24 directions. Other parameters were: TR/TE=2000/13.5 ms, b-value 500-700 s/mm^2^ and isotropic resolution of 280 μm. The human and sheep hearts were imaged on a clinical 3T Siemens scanner with an isotropic resolution of 2 mm^3^, TR/TE=8430/96 ms, and a b-value of 2000 s/mm^2^. The tractographic propagation angle PA was defined as the angle between two adjacent principal eigenvectors (ê_ij_, ê_ij+1_) relative to a given fiber (Figure 1A). PA values were computed along myofiber trajectories within the principal eigenvector field using a 4^th^ order Runge-Kutta integration method. Gd-DE imaging was performed 10min after the injection of 0.2mmol Gd-DTPA/kg. A short axis slice through the infarcted myocardium was acquired using a cardiac-gated inversion recovery gradient echo sequence. Infarcted regions were segmented automatically on the Gd-DE images using a threshold of 2 standard deviations above normal. A PA threshold value greater than 4 degrees was used to automatically segment infarcted myocardium. Percent infarct size was calculated with both techniques and correlated.

## Results

**Figure 2:**
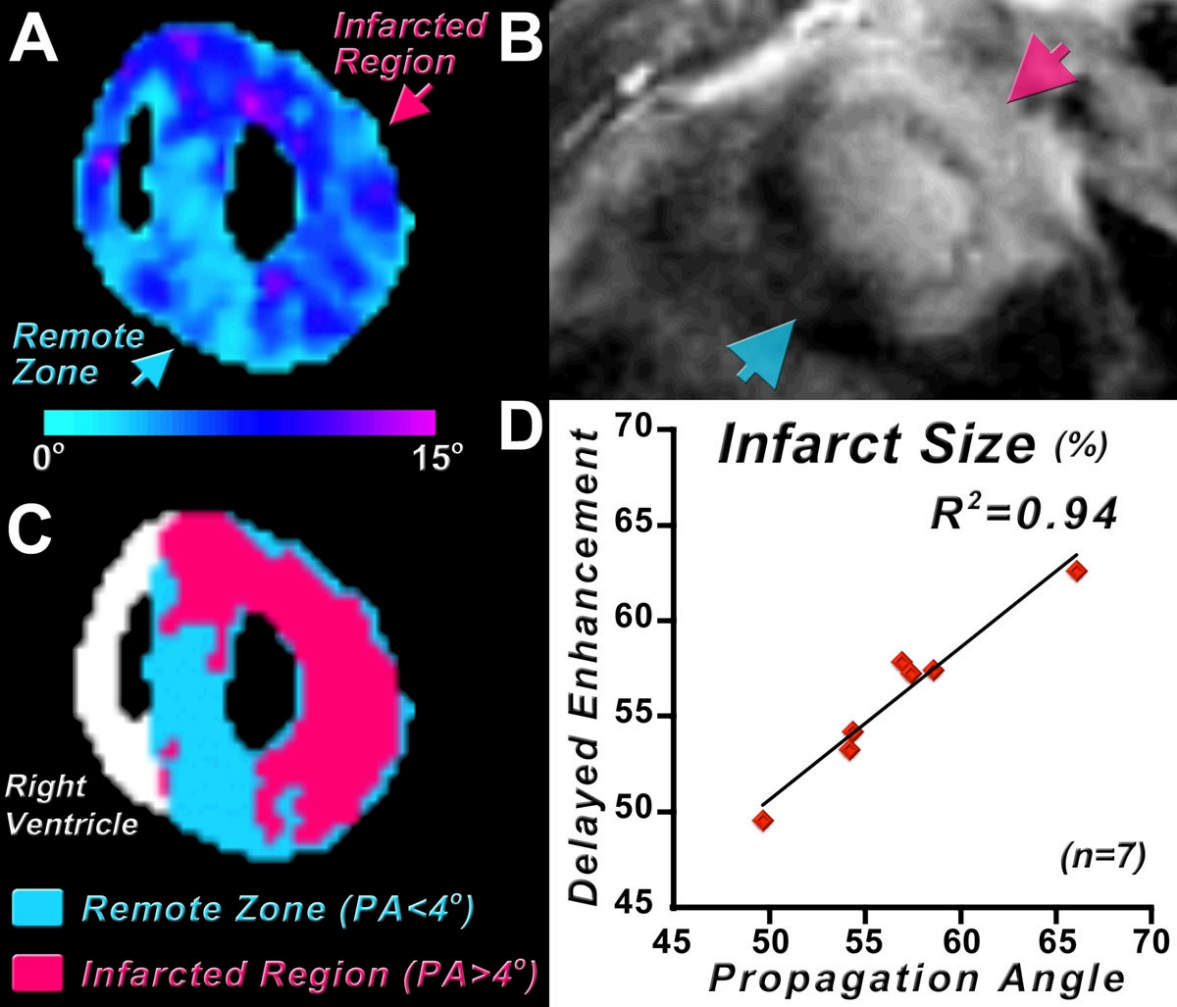
*In vivo* PA maps in infarcted mice. (A) PA map in a mouse with a large anterolateral infarct. (B) Delayed enhancement image at the corresponding level. It should be noted that the PA maps were acquired in mid-systole and the delayed enhancement images in mid-diastole. (C) Segmentation of the PA map using a threshold value of 4 degrees robustly segments normal from infarcted myocardium. (D) A high correlation (R^2^=0.94) between infarct size calculated from the *In vivo* PA and infarct size measured by delayed gadolinium enhancement was obtained.

Tractography of a normal human heart color-coded by the PA is shown in Figure 1B. PA in the normal myocardium is highly homogeneous, averaging between 2 and 4 degrees. PA in the sheep infarct is significantly elevated and allows the infarct zone to be differentiated from the rest of the myocardium (Figure 1 C-D). Both PA (Figure 2A) and Gd-DE uptake (Figure 2B) were significantly increased in the infarct zone of all the mouse hearts imaged. A PA threshold of 4 degrees robustly segmented the infarct zone (Figure 2C), and an excellent correlation (R^2^=0.94) was seen between percent infarct size by Gd-DE and tractographic PA (Figure 2D).

## Conclusions

PA detects the loss of tract coherence in infarcted myocardium and robustly delineates myocardial infarcts *in vivo*. The use of DTI, and hence the tractographic PA, does not require exogenous contrast and can be performed in all patients regardless of renal function. The technique provides a complementary and valuable adjunct to Gd-DE.

## Funding

R01HL093038
